# Transgenic overexpression of NanogP8 in the mouse prostate is insufficient to initiate tumorigenesis but weakly promotes tumor development in the Hi-Myc mouse model

**DOI:** 10.18632/oncotarget.17186

**Published:** 2017-04-18

**Authors:** Bigang Liu, Shuai Gong, Qiuhui Li, Xin Chen, John Moore, Mahipal V. Suraneni, Mark D. Badeaux, Collene R. Jeter, Jianjun Shen, Rashid Mehmood, Qingxia Fan, Dean G. Tang

**Affiliations:** ^1^ Department of Molecular Carcinogenesis, University of Texas M.D Anderson Cancer Center, Science Park, Smithville, TX 78957, USA; ^2^ Department of Oncology, The First Affiliated Hospital of Zhengzhou University, Zhengzhou, Henan 450052, China; ^3^ Department of Pharmacology & Therapeutics, Roswell Park Cancer Institute, Buffalo, NY 14263, USA; ^4^ Cancer Stem Cell Institute, Research Center for Translational Medicine, East Hospital, Tongji University School of Medicine, Shanghai 200120, China

**Keywords:** NanogP8, Nanog, prostate, prostate cancer, stem cells

## Abstract

This project was undertaken to address a critical cancer biology question: Is overexpression of the pluripotency molecule Nanog sufficient to initiate tumor development in a somatic tissue? Nanog1 is critical for the self-renewal and pluripotency of ES cells, and its retrotransposed homolog, NanogP8 is preferentially expressed in somatic cancer cells. Our work has shown that shRNA-mediated knockdown of NanogP8 in prostate, breast, and colon cancer cells inhibits tumor regeneration whereas inducible overexpression of NanogP8 promotes cancer stem cell phenotypes and properties. To address the key unanswered question whether tissue-specific overexpression of NanogP8 is sufficient to promote tumor development *in vivo*, we generated a NanogP8 transgenic mouse model, in which the ARR_2_PB promoter was used to drive NanogP8 cDNA. Surprisingly, the ARR_2_PB-NanogP8 transgenic mice were viable, developed normally, and did not form spontaneous tumors in >2 years. Also, both wild type and ARR_2_PB-NanogP8 transgenic mice responded similarly to castration and regeneration and castrated ARR_2_PB-NanogP8 transgenic mice also did not develop tumors. By crossing the ARR_2_PB-NanogP8 transgenic mice with ARR_2_PB-Myc (i.e., Hi-Myc) mice, we found that the double transgenic (i.e., ARR_2_PB-NanogP8; Hi-Myc) mice showed similar tumor incidence and histology to the Hi-Myc mice. Interestingly, however, we observed white dots in the ventral lobes of the double transgenic prostates, which were characterized as overgrown ductules/buds featured by crowded atypical Nanog-expressing luminal cells. Taken together, our present work demonstrates that transgenic overexpression of NanogP8 in the mouse prostate is insufficient to initiate tumorigenesis but weakly promotes tumor development in the Hi-Myc mouse model.

## INTRODUCTION

Prostate cancer (PCa) is the most common cancer type and the second leading cause of cancer-associated death for males in Europe and the United States [1, 2]. Androgen deprivation therapy (ADT) remains the mainstay of treatment for advanced PCa patients [3]. The majority of treated patients initially responds well to ADT but eventually develops resistance and progresses to the more aggressive form of PCa defined as castration-resistant prostate cancer (CRPC) [4]. As CRPC progression is associated with increased incidence of metastatic dissemination and patient death [5], CRPC is currently incurable. It is critical to understand molecular mechanisms underlying PCa development/progression and therapy resistance.

Nanog (also called Nanog1) is a core pluripotency transcription factor in embryonic stem (ES) cells. Its retrotransposed homolog NanogP8 has been reported to be expressed in a variety of cancers, and their expression levels have been positively correlated with poor survival of cancer patients [6–34]. Our lab has shown that various cancer cells preferentially express NanogP8 mRNA, primary PCa samples contain more Nanog protein-expressing cells than the matched benign tissues, and down-regulation of endogenous Nanog inhibits tumor regeneration in prostate, breast and colon cancer cells [35]. We have further shown that NanogP8-expressing cancer cells possess cancer stem cell (CSC) properties and inducible expression of NanogP8 in bulk PCa cells promotes the acquisition of CSC and CRPC properties [36], implying that NanogP8 might play a functional role in PCa progression to the CRPC state.

Despite the solid evidence that NanogP8 promotes the defined characteristics of CSCs [9, 35–37] and functions as an oncogenic factor *in vitro*, it remains unclear whether NanogP8 might exhibit pro-tumorigenic activities *in vivo*, and, in particular, whether tissue-specific overexpression of NanogP8 is sufficient to promotes prostate tumorigenesis. We initially hypothesized that similar to Oct4 and Sox2 overexpression [38, 39], NanogP8 expression in epithelial cells might be able to trigger spontaneous tumor formation in mice. Therefore, we developed a NanogP8 transgenic mouse model in which NanogP8 cDNA was driven by a cytokeratin 14 (K14) promoter that directs NanogP8 expression in cytokeratin 14 cellular compartments including the basal cells of the prostate and skin [40]. The K14-NanogP8 animals, however, did not develop spontaneous tumors in any organs, and, even more surprisingly, the animals of the transgenic line with a high level of NanogP8 expression actually exhibited reduced tumor development in a 2-stage chemical carcinogenesis setting as a consequence of the depletion of keratinocyte stem cells [40].

To directly address whether transgenic expression of NanogP8 is sufficient to initiate prostate tumorigenesis, in this study, we generated an ARR_2_PB-NanogP8 transgenic mouse model to direct NanogP8 expression in luminal cells of the mouse prostate. Our results indicate that overexpression of NanogP8 alone in prostate luminal cells is unable to initiate mouse prostate tumor development in both androgen intact and androgen-deficient conditions, although NanogP8 expression appears to slightly promote prostate tumorigenesis in ARR_2_PB-Myc mice.

## RESULTS

### Generation and characterization of ARR_2_PB-NanogP8 transgenic mouse model

Prostate epithelia are mainly composed of three cell types, namely, basal, luminal, and neuroendocrine cells [41]. It is well recognized that the prostatic basal cells are less differentiated than luminal cells [42] and that some subpopulations of basal cells exhibit stem cell characteristics [43–45]. Consequently, prostate basal cells have once been reported as the preferred cellular origin for cancer. However, our CK14-NanogP8 transgenic mice did not develop spontaneous tumors in the prostate [40], suggesting that over-expression of NanogP8 in basal cells is not sufficient to initiate prostate tumorigenesis.

**Figure 1:**
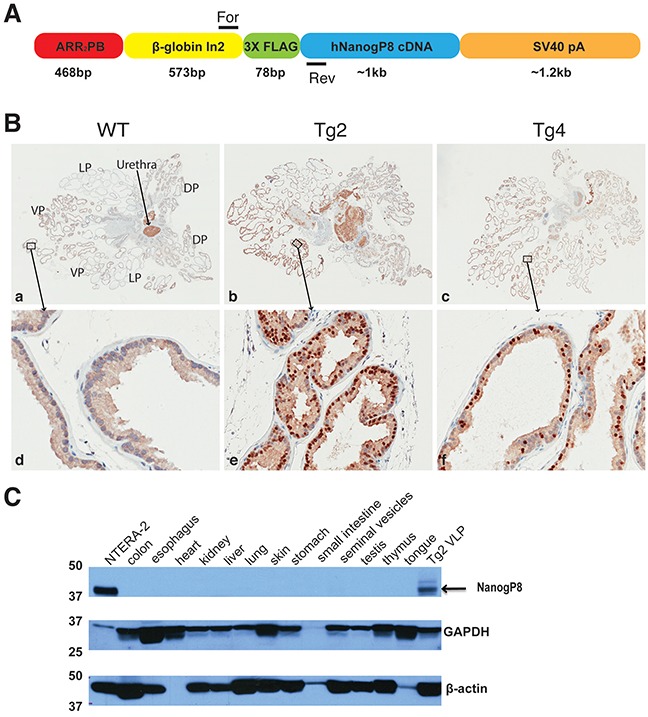
The generation and characterization of ARR2PB-NanogP8 mice **(A)** Schematic of the ARR_2_PB-NanogP8 transgene construct. A 3× Flag tagged human NanogP8 (hNanogP8) cDNA was put under the control of ARR_2_PB promoter. β-globin In2 denotes rabbit β-globin second intron sequence. For and Rev denote the forward and reverse primers for genotyping. The lengths for each modular element in the construct are indicated below. **(B)** NanogP8 protein expression was analyzed byIHC analysis (using a goat pAb against Nanog; R & D, AF1997) in the whole-mount prostates of WT, Tg2, and Tg4 mice. **(a-c)** Representative low-magnification images (40x) of whole-mount prostate sections. Boxed areas are located in the ventral prostate lobes, which are enlarged and shown in panels (**d-f**; below). The orientation of the whole-mount images is illustrated in panel **(a)**. Dark brown nuclear stain indicates NanogP8 positive cells whereas blue color indicates nuclear counterstaining. **(C)** Western blotting analysis with an anti-Nanog antibody (Cell Signaling) was used to determine the expression of NanogP8 in different organs/tissues of adult transgenic mouse Tg2 (8 weeks). Nanog protein from embryonic carcinoma NTERA-2 cells was used as the positive control (upper panel). GAPDH and β-actin were used as loading controls.

Inspired by recent findings that prostate luminal cells can serve as the cell-of-origin for both primary and castration-resistant PCa [46] and that a rare castration-resistant luminal prostate cell population possesses multipotent stem cell activity [47], we utilized an ARR_2_PB promoter to drive the expression of a human NanogP8 cDNA tagged with 3X Flag (Figure 1A) to generate a transgenic mouse model in which NanogP8 was specifically expressed in prostatic luminal cells. The ARR_2_PB promoter, developed from the rat probasin promoter, contained two androgen-responsive regions (ARRs), which confers high levels of gene expression in prostatic luminal cells of transgenic mice [48, 49]. We obtained five potential founders, of which only line 2 and 4 showed the germ-line transmission. The transgenic (Tg) animals from these two lines were fertile and developed normally, and we did not observe any visible phenotypes compared with their wild type (WT) littermates. Immunohistochemistry (IHC) analysis with an anti-Nanog antibody revealed that most of Nanog-positive cells were the luminal cells and that Nanog expression was the highest in lateral lobe (LP) and ventral lobes (VP) followed by dorsal lobes (DP) whereas only scattered Nanog-positive cells were detected in the anterior prostate (AP). This expression pattern of transgene NanogP8 was very similar to those of other ARR_2_PB promoter-driven transgenes [49–52]. IHC staining also demonstrated that the transgenic expression level of NanogP8 in transgenic line 2 (Tg2) prostates was much higher than that of transgenic line 4 (Tg4) prostates (Figure 1B). We therefore utilized Tg2 mice in most subsequent experiments. Western blot analysis with different tissue/organs from Tg2 mice detected the Nanog band (∼42 kD; the lysate of embryonal carcinoma NTERA-2 cells was loaded as a positive control for Nanog protein) only in the prostate tissue (Figure 1C). The above results suggest that we have successfully established a NanogP8 transgenic mouse model in which NanogP8 is specifically expressed in the prostate tissue of the transgenic animals.

### Overexpression of NanogP8 does not affect mouse prostate development nor induces spontaneous tumor formation

**Figure 2:**
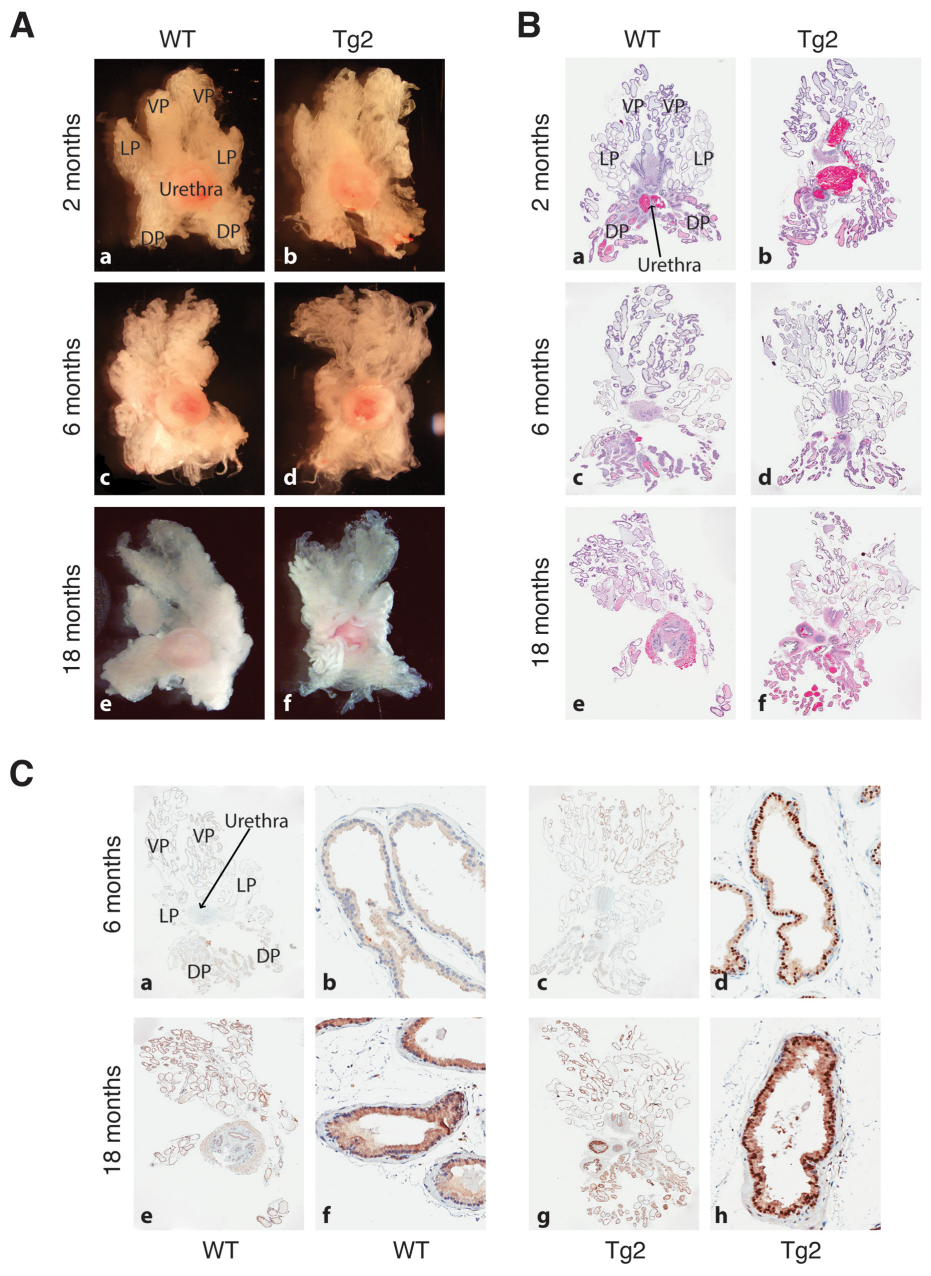
Prostate morphologies in WT and Tg2 mice at different ages **(A)** Representative images of microdissected prostates in WT and transgenic mice at 3 different ages. **(B)** Representative H&E images of whole-mount prostate sections of WT and Tg2 mice at 3 ages. **(C)** Representative IHC images of NanogP8 staining in whole-mount prostate sections in WT and transgenic mice at 6 and 18 months of age. Shown on the left (panels **a, c, e, g**) are low-mag images (40×) and on the right ventral lobes at high magnifications (200×; panels **b, d, f, h**).

We isolated and microdissected prostates and performed whole-mount organ analysis from WT and Tg2 animals (n>30 for each) at the age of 2, 6, and 18 months, respectively. The Tg2 prostates displayed similar morphologies and sizes to their age-matched WT prostates (Figure 2A). Hematoxylin and eosin (H&E) staining also revealed that the histological structures of prostate glands in WT and Tg2 mice were indistinguishable at all ages analyzed (Figure 2B). We confirmed that the NanogP8 protein was consistently expressed in the nuclei of luminal cells in Tg2 prostates, particularly in their VPs and LPs (Figure 2C). By continuously monitoring a cohort of Tg (n>60) and WT (n>40) mice for up to 2 years, we did not observe any spontaneous tumors, and histological examination did not detect any obvious hyperplasia or prostatic intraepithelial neoplasia (PIN) in the prostate tissues (Figure 2B) or any abnormalities in multiple other tissues /organs (Supplementary Figure 1) in mice of both genotypes. In addition, there were no statistically significant differences in animal life spans and body weights, and the prostate sizes between Tg2 and WT mice during the 2 years of observation period (data not shown). Our results indicate that NanogP8 overexpression does not affect mouse prostate development and is insufficient to induce prostate tumorigenesis.

### Transgenic NanogP8 expression in prostatic luminal cells is incapable of initiating tumors in castrated mouse prostates

**Figure 3:**
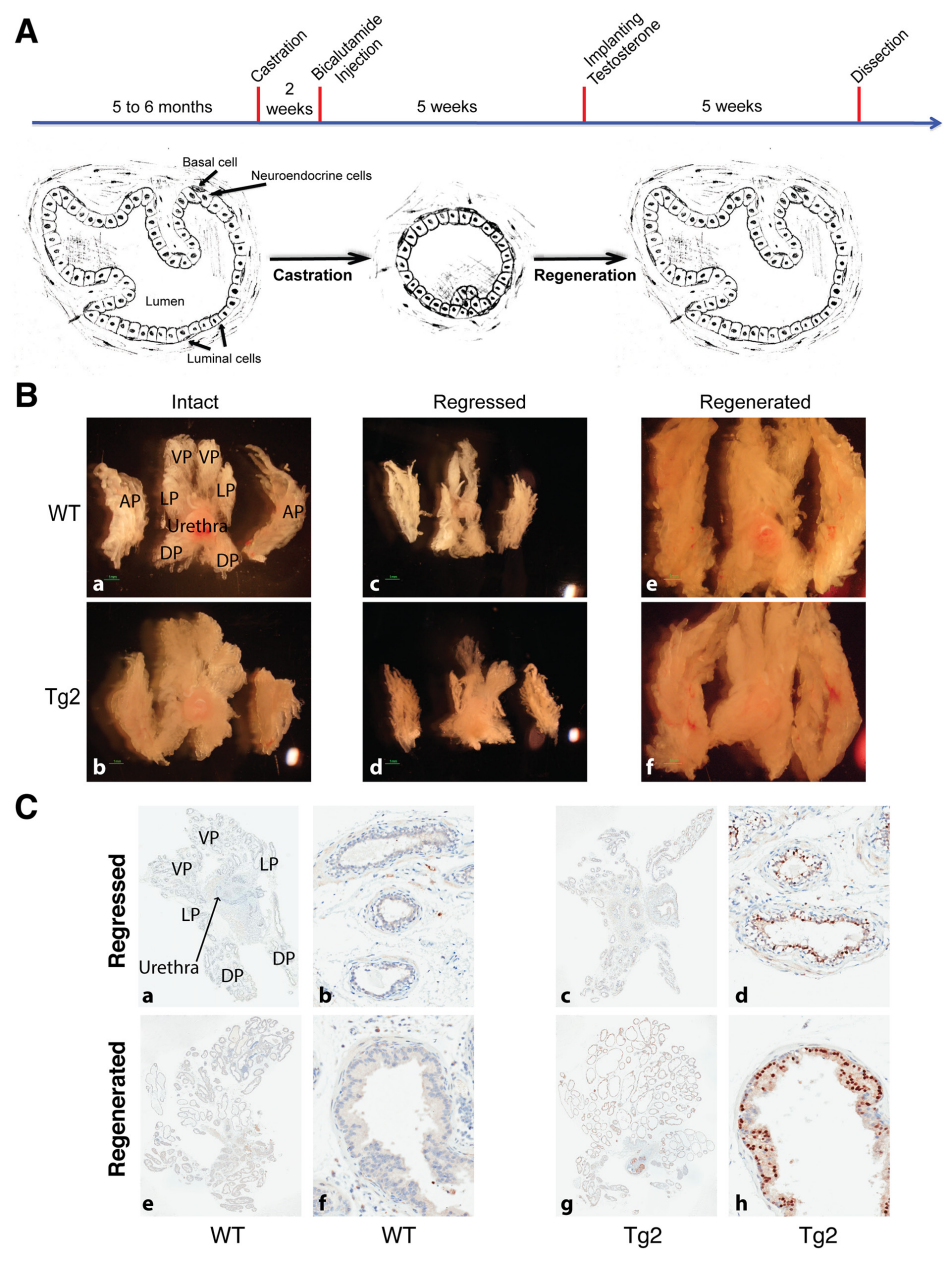
Overexpression of NanogP8 does not promote mouse prostate growth in castrated conditions **(A)** A schematic representation of mouse prostate duct in intact, regressed, and regenerated conditions. After castration, the mice were injected with bicalutamide (5 mg/kg body weight/injection) for 5 weeks (twice/week) to fully suppress the activity of androgen receptor. Most luminal cells died from apoptosis but most basal cells survived. Upon the re-administration of testosterone, regressed prostates initiate the regeneration process and restore its histological structure. **(B)** Representative images of whole-mount prostate morphologies in WT and transgenic mice at the intact, regressed, and regenerated conditions. **(C)** Representative IHC images of NanogP8 staining in WT (left) and Tg2 (right) whole-mount prostate sections. The 4 prostate lobes were indicated on the upper left panel. Panels a, c, e, and g represent low magnification (40x) whereas panels b, d, f, and h are high magnification (200x) images.

The prostate is dependent upon androgens to maintain its structure and function [53]. In androgen-deprived conditions such as surgical castration or administration of inhibitors of androgen synthesis and/or androgen receptor, the prostate gland undergoes rapid regression to an atrophic state because most luminal cells die from apoptosis [41, 54]. Upon re-administration of androgen to the castrated mice, the atrophic prostate can rapidly restore to its pre-castrate size and functions [41, 55, 56]. We have shown that enforced expression of NanogP8 promotes prostate CSC characteristics and, in xenograft mouse models, NanogP8 overexpression promotes prostate tumor growth in castrated conditions [36]. These findings led us to hypothesize that overexpression of NanogP8 in the mouse prostate may help maintain and further stimulate the growth of prostatic ducts during the regression-regeneration cycles. We tested this hypothesis by performing surgical castration and regeneration experiments (Figure 3A). In addition to testis, adrenal cortex also produces a small amount of androgens [57]. Therefore, two weeks following surgical castration, we injected the castrated mice with bicalutamide, an androgen receptor inhibitor, at a dose of 5 mg/kg body weights, twice a week for 5 weeks, to fully deplete androgens. Five weeks after castration, we carried out microdissection analysis in some animals (five mice for each group). We observed that the morphologies and sizes of prostate glands from both WT and Tg2 mice were dramatically reduced and shrunk, and the survived ducts were in an atrophic condition and fibrosis appeared in all lobes, indicating regression occurred in prostate glands (Figure 3B). Overall, we did not observe obvious differences in gross morphology of prostate glands between the two types of mice (Figure 3B–3C). IHC analysis with an anti-Nanog antibody detected fewer and weaker Nanog-positive cells in the atrophic prostates in Tg2 mice than in the regenerated prostates of Tg2 mice (Figure 3C), which is related to the regulation of ARR_2_PB promoter and the transgene by androgen.

**Figure 4:**
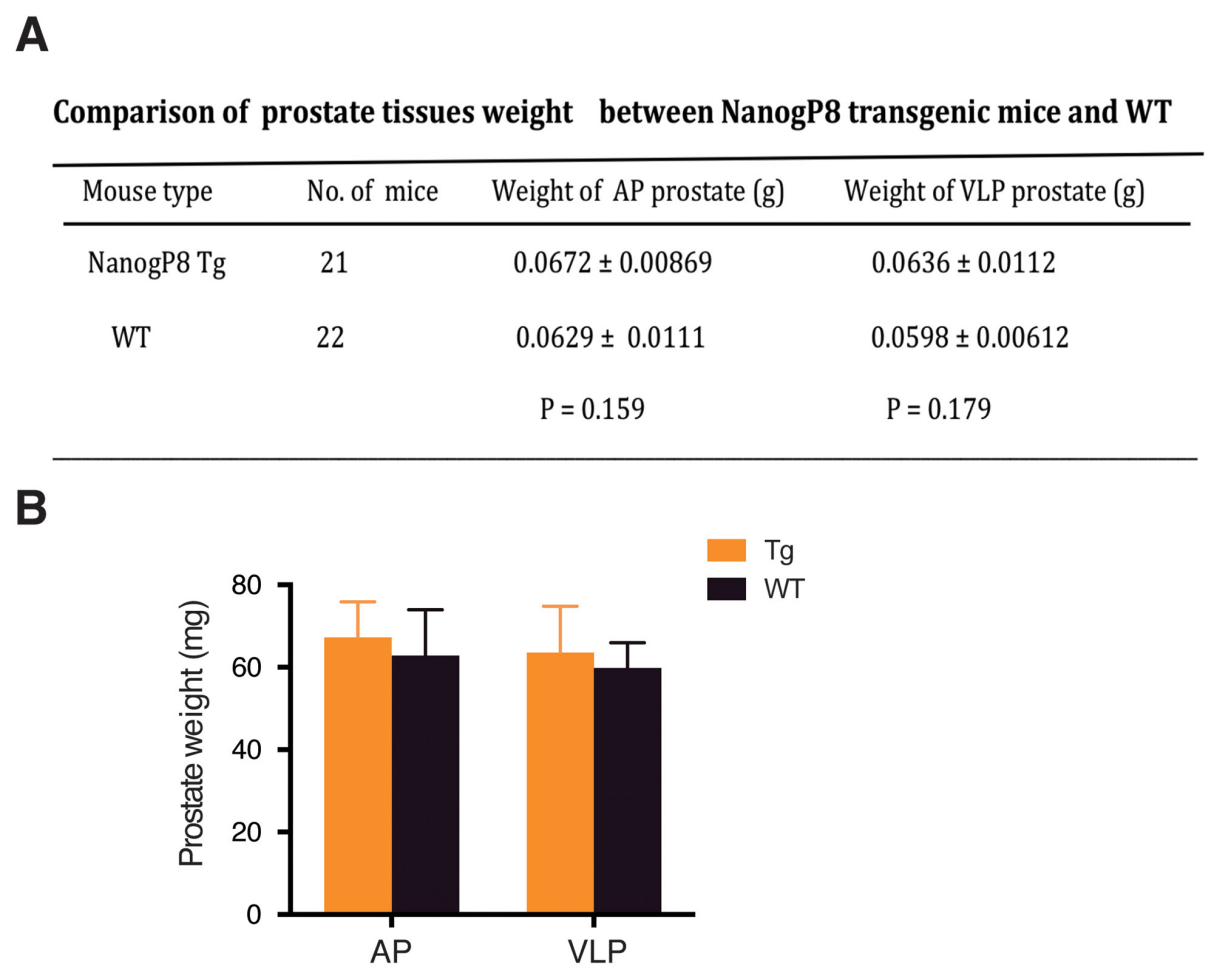
Similar prostate weights between WT and transgenic mice Table **(A)** and bar graph **(B)** presentations of the wet weights of AP and VLP lobes in WT and Tg prostates.

Subsequently, we implanted a testosterone pellet into the dorsal skin in cohorts of mice (WT, n=21; Tg2, n=22) to initiate regeneration and 5 weeks later, prostates were isolated for whole-mount analysis. We observed that, upon androgen replacement, prostate glands of the castrated mice recovered normal morphologies and sizes from the atrophic status (Figure 3B). H&E staining revealed that the two types of mice displayed the normal structures in ducts and branches in their regenerated prostate glands (data not shown). Nanog-positive cells were luminal cells and cell numbers were comparable to those in the non-castrated organs (Figure 3C). In the course of castration-regeneration experiments, we did not observe any prostate hyperplasia, PIN and spontaneous tumor in WT and Tg2 mice (not shown). Contrary to our expectations, the gross phenotypes and histological structures of prostate glands were very similar between the two groups (Figure 3B–3C) and transgenic expression of NanogP8 in mouse prostate did not appreciably increase cell proliferation of the prostate glands during regeneration (not shown). Additionally, the weights of VLPs (ventral/lateral prostate lobes) and APs of the regenerated prostates between the two groups were similar (Figure 4).

Collectively, the above findings suggest that expression of NanogP8 in luminal cells of the mouse prostate gland is unable to initiate tumorigenesis in castrated conditions.

### Evidence that NanogP8 accelerates prostate tumorigenesis in ARR_2_PB-Myc (Hi-Myc) mice

**Figure 5:**
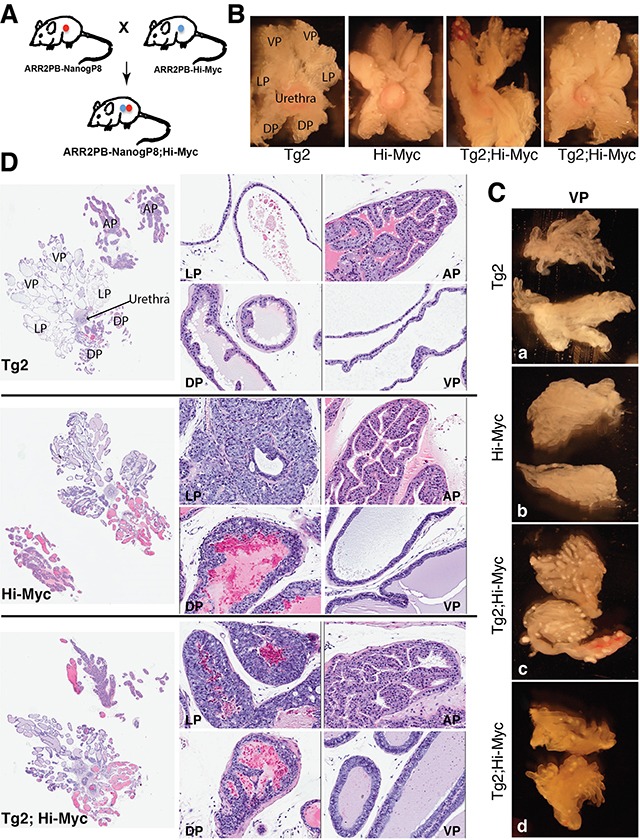
Gross morphological and histological changes in Tg2; Hi-Myc double transgenic prostates **(A)** The strategy of generating double transgenic mice. **(B)** Representative images of microdissected whole-mount prostates from Tg2, Hi-Myc, and double transgenic mice of 3 months. Note that the white dots were observed only in the ventral lobes (VPs) of the double transgenic mice. **(C)** Representative images of microdissected VPs from 2-month old Tg2 **(a)**, Hi-Myc **(b)**, and Tg2; Hi-Myc (**c** and **d**; 2 animals) transgenic mice. Note prominent white spots only in the Tg2; Hi-Myc prostates. **(D)** H&E staining of whole-mount prostate sections of 3-month old Tg2, Hi-Myc, and double transgenic mice. The left panels are whole-mount prostate sections with low magnifications (40×); the middle and right panels are the four lobes of prostate sections (LP, DP, AP, and VP) with high magnifications (200×).

A recent study demonstrated that NanogP8 expression alone in the mammary tissue is incapable of inducing mammary tumors but could enhance mammary tumorigenesis and accelerate metastasis of Wnt-1 transgenic mice [58]. Therefore, we decided to examine whether NanogP8 might cooperate with other oncogenic factors to regulate the progression of PCa. Myc is overexpressed in >80% human PCa due to genomic amplification and post-transcriptional mechanisms [59–61] and forced expression of Myc in mouse prostates in Hi-Myc transgenic mice reliably leads to the development of murine PIN that progresses to invasive adenocarcinomas at 3 - 6 months age [51]. Thus, we crossed Hi-Myc transgenic mice with our Tg2 (ARR_2_PB-NanogP8) mice to generate the Tg2; Hi-Myc double transgenic mice (Figure 5A). By IHC analysis (Supplementary Figure 2), we confirmed that both Nanog and Myc proteins were expressed in the luminal cells of Tg2; Hi-Myc prostates. With cohorts of Tg2 (n>20), Hi-Myc (n>20), and Tg2; Hi-Myc (n>30) mice observed side-by-side for >1 year, the Tg2; Hi-Myc mice behaved normally and were fertile, and when compared with age-matched Hi-Myc mice, the Tg2; Hi-Myc mice did not display any visibly different phenotypes and showed similar lifespan.

We performed microdissection analysis at different time points, starting from 2 months to one year (n>20 for each group) and compared the gross appearance and structures of the prostates in Tg2; Hi-Myc mice versus Hi-Myc mice. Morphologically, the gross phenotypes of the whole-mount prostate lobes from Tg2;Hi-Myc mice were indistinguishable from those of the Hi-Myc mice, both of which manifested clear-cut hyperplasia (i.e., PIN or prostate intraepithelial neoplasia) and tumor growth (Figure 5B). Very interestingly, however, we observed numerous white dots in most (>80%) of the VPs in Tg2; Hi-Myc double transgenic but not Hi-Myc mice (Figure 5B). The prominent white dots, which appeared as early as two months of age, were confirmed in the microdissected VP lobes (Figure 5C).

All mice from Tg2; Hi-Myc and Hi-Myc groups (∼3 months) developed multiple PINs and adenocarcinomas in their prostate glands and H&E staining indicated that the histological features of AP and DLP (dorsal and lateral prostate) lobes in the two groups of mice were overall similar (Figure 5D). These histological features represented the typical lesions of the Hi-Myc prostates [51] with numerous late-stage PIN foci detected in the LP and VP ducts (Figure 5D; data not shown). These PINs were characterized by multifocal proliferative lesions of atypical epithelial cells within preexisting ducts and acini, with the foci varying in the number of cell layers and the degree and pattern of atypia. Generally, atypical cells of the PIN in the LP, which were poorly oriented and almost filled the lumen, showed large irregular nuclei, and exhibited hyperchromatic or vesicular chromatin patterns with prominent nucleoli (Figure 5D). Extensive and severe hyperplasia were also detected in all ducts of the VP, and the hyperplastic areas contained crowded cells, which displayed nuclear atypia with nuclear enlargement and prominent nucleoli (Figure 5D). In contrast to the pathologies in the DLPs, we did not observe any apparent lesions in the APs of Hi-Myc and Tg2; Hi-Myc mice of 3-6 months (Figure 5D; data not shown). As the control, all Tg2 mice did not develop any hyperplasia, PIN, or adenocarcinoma during the entire experimental period (>1 year) and their prostate glands also showed normal morphology (Figure 5D; data not shown).

**Figure 6:**
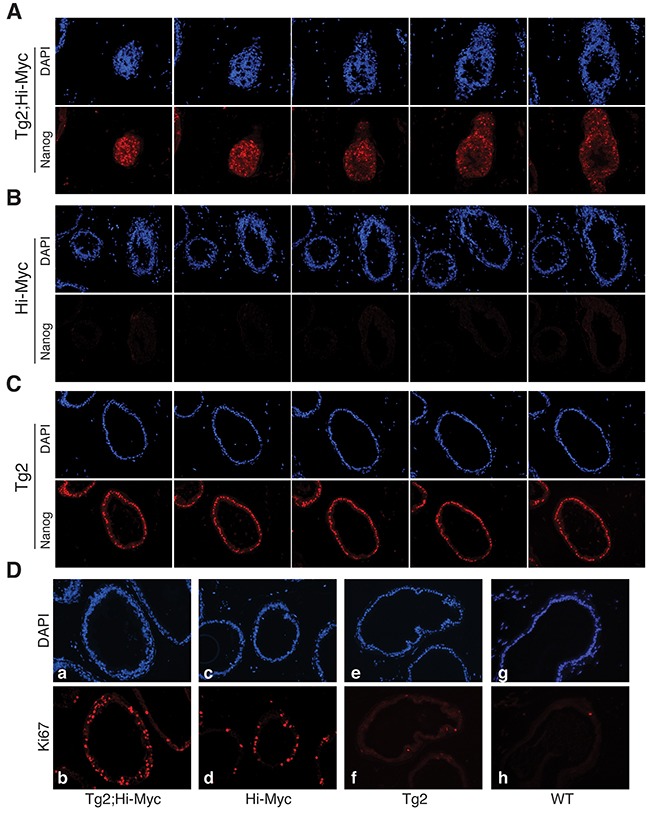
The prominent white spots in Tg2; Hi-Myc VPs represent clusters of Nanog-expressing cells **(A-C)** Nanog and DAPI immunofluorescence analysis in serial paraffin sections of VPs from the three genotypes of transgenic mice (∼3 months of age). The immunofluorescence staining of DAPI (upper panels) and Nanog (lower panels) of five serial VP sections of the Tg2; Hi-Myc double transgenic mice revealed clusters of crowded Nanog-expressing cells. **(D)** Immunofluorescence of DAPI (upper panels) and Ki67 (lower panels) in the VPs from the mice indicated.

**Figure 7:**
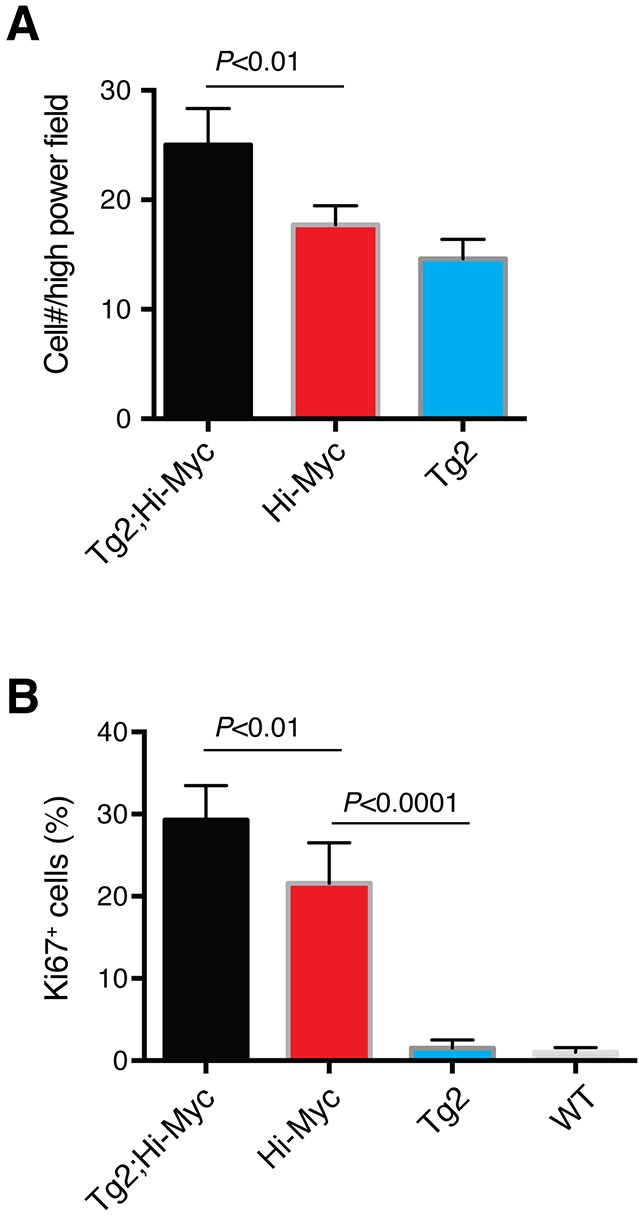
Quantification of proliferation (i.e., Ki67^+^ cells) in the VP lobes of different mice **(A)** Bar graph presentation of cell numbers per 200x microscopic field in the VPs of three genotypes of mice. **(B)** Bar graph presentation of the % of Ki67^+^ cells in the VPs of 3 genotypes of mice.

Of interest, the VP ducts in the double transgenic mice displayed more severe hyperplasia with apparently thicker epithelial layers containing more atypical cells compared to the VP ducts in Hi-Myc mice (Figure 5D). Also, the VPs of the double transgenic mice were the only prostate lobes that developed prominent white spots (Figure 5B–5C). Consequently, we microdissected out the VPs (Figure 5C) from the whole-mount prostates to carry out immunofluorescence staining of Nanog on serial cryosections. Remarkably, we observed many overgrown ‘ductules’ or ‘buds’ with Nanog-positive cells in the VPs of Tg2; Hi-Myc but not Hi-Myc mice (Figure 6A–6B). These ductules/buds were crowded with numerous atypical cells that filled almost half of the lumens (Figure 6A). We thus interpret that the white dots in the VPs of double transgenic mice are the newly overgrown ductules/buds from Nanog-expressing cells. Notably, most ducts in the Tg2; Hi-Myc VPs had two or more cell layers that were crowded with atypical cells whereas the majority of ducts in the VPs of Hi-Myc mice only contained one cell layer (Figure 6A–6B and data not shown). Counting the cell numbers of epithelia in 10 microscopic fields showed that the Tg2; Hi-Myc VPs had significantly higher cellularity than the Hi-Myc VPs (Figure 6D; Figure 7A). Immunofluorescence staining with an anti-Ki67 antibody confirmed that the VPs of the double transgenic mice contained more Ki67-positive cells than the Hi-Myc VPs (Figure 7B).

## DISCUSSION

Our lab has generated a substantial body of evidence showing the potential oncogenic functions of Nanog in somatic human cancer cells, in particular, PCa cells [35, 36, 40, 62–66]. Thus, knocking down endogenous Nanog in several cancer cell types inhibits xenograft tumor regeneration and growth in immunodeficient mice [35] whereas inducible expression of a Nanog transgene in cancer cells promotes xenograft tumor development as well as transition from androgen-dependent to androgen-independent PCa [36]. Moreover, knocking down endogenous Nanog in the cancer stem cell-enriched PSA^−/lo^ cell population in the LAPC9 model greatly dampens their tumor-regenerating activity in castrated hosts [64]. In fact, we have recently shown that castration upregulates Nanog expression, which is in turn required for the regeneration and maintenance of androgen-independent PCa [66]. These xenograft studies [35, 36, 64, 66] made us to expect some Nanog-associated tumor phenotypes in transgenic animal models. Thus, it came to us as a surprise that overexpression of NanogP8 in K14-expressing basal cells, including prostatic basal cells, does not initiate spontaneous tumors in any organs including the prostate [40]. Results from the present study indicate that even targeted overexpression of NanogP8 specifically in the luminal cells of the mouse prostate does not, by itself, promote tumor development. These two studies of ours suggest that NanogP8 overexpression alone is insufficient to initiate tumorigenesis in somatic tissues/organs including the skin and prostate. This conclusion is consistent with two other studies showing that NanogP8 overexpression alone in transgenic animals is unable to initiate mammary tumor development [58] or Nanog only weakly enhances liver tumorigenesis in a hepatocellular carcinoma reconstitution model [67].

There exists a possibility that the oncogenic functions of Nanog *in vivo* require cooperation from other oncogenes in different contexts. Indeed, transgenic expression of Nanog, though insufficient to initiate tumor development, promotes β-catenin-induced mammary tumorigenesis and metastasis [62]. In the present study, we have also observed that prostate-specific NanogP8 overexpression slightly accelerates the tumorigenic process in the VPs of Hi-Myc mice, suggesting that Nanog might cooperate with Myc in promoting tumor development. In this regard, we have presented evidence that Nanog and Myc may form a feed-forward regulatory loop (i.e., they positively regulate each other) in PCa cells [36, 66] suggesting that these two master transcription factors may overlap significantly in their signaling pathways, explaining why the phenotypes in the compound mice are relatively subtle. This raises the possibility that crossing our NanogP8 line with other Tg models in which the oncogenic signaling differs significantly from Nanog might more dramatically enhance tumor development and progression. We are currently testing this possibility in several different mouse models.

## MATERIALS AND METHODS

### Generation and genotyping of ARR_2_PB-NanogP8 transgenic mice

Basic procedures for establishing Tg animals have been described [40, 50, 68]. A 3X Flag tagged NanogP8 cDNA derived from a patient primary prostate tumor was subcloned into the multiple cloning site of pPB.197 vector that contains a rat ARR_2_PB promoter [48], and the construct (ARR_2_PB-NanogP8) was used to generate ARR_2_PB-NanogP8 mice with FVB background at the Transgenic Core in our department. For genotyping, mouse tail snips were collected and lysed in the solution containing 100 mM Tris-HCl (pH 8.0), 200 mM NaCl, 5 mM EDTA, 0.5% SDS and 0.2 mg/ml proteinase K at 55°C overnight. β-globin forward (For) primer 5′-GGGCAACGTGCTGGTTAT-3′and NanogP8 reverse (Rev) primer 5′-CCTTTGGGACTGGTGGAA-3′ (see Figure 1A) were used in PCR to generate a ∼300 bp fragment to identify the transgenic mice.

### Prostate microdissection and isolation of whole-mount prostate

Detailed procedure was described in earlier publications by Sugimura et al [54, 69]. Briefly, after sacrificing mice, the prostates were removed along with the urogenital tract. The prostates were placed immediately in ice-cold phosphate-buffered saline (PBS) and microdissected under a dissection microscope to remove fat and collective tissues. The isolated whole-mount prostates were photographed by Nikon digital camera (DXM1200F) and then put into 10% formalin for histological analysis.

### Harvest of murine organs

After sacrificing mice, the internal organs and skin samples were removed quickly and placed directly into microcentrifuge tubes, and then were immersed in liquid nitrogen for cryopulverizing. The organ powders were lysed in chilled RIPA buffer containing protease inhibitor cocktail (1:100, Sigma). The lysates were centrifuged at 16,000 g for 20′ at 4°C and the supernatants were used for Western blot. Alternatively, the organs were placed into cassettes and immersed in 10% formalin for 24-48 h for immunohistochemistry.

### Antibodies used, western blot analysis, immunohistochemistry (IHC), and immunofluorescence

A rabbit monoclonal antibody (mAb) against Nanog (clone D73G4, Cell Signaling, cat. # 4903) was used in Western blotting. A goat pAb against Nanog (R & D, AF1997) was used in IHC and immunofluorescence staining. Two other antibodies used in this study were a rabbit mAb to c-Myc (clone EP121; Epitomics, cat. #1472-1) and a rabbit pAb to Ki-67 (Leica Biosystems, cat. # NCL-Ki67p). For Western analysis, 80 μg protein samples were analyzed by 12.5% SDS-PAGE and gels were transferred onto an Immobilon-P transfer membrane (PVDF, Millipore, Bedford, MA). The membrane was blocked with 5% non-fat dried milk in TBST (10 mM Tris-HCl, 150 mM NaCl and 0.1% Tween-20) for 1 h at room temperature, and incubated overnight at 4°C with an anti-Nanog antibody. Membranes were washed three times with TBST buffer, then incubated for 1 h with 1:2000 secondary antibodies, and developed with ECL Plus WB detection reagent (PerkinElmer). β-actin (Sigma, St. Louis, MO) and GDPAH (Santa Cruz) antibodies were used as the loading control. IHC and immunofluorescence staining were performed as previously descripted [70, 71].

### Castration and regeneration of the mouse prostates

Castration was carried out using standard techniques [54]. Briefly, mice were anesthetized by the intraperitonealinjection of ketamine. Castrations were performed by complete removal of the testes and epididymis through a scrotal approach. The distal end of spermatic cord was ligated with surgical suture. To fully suppress androgen activity, two weeks later, bicalutamide at a dose of 5 mg/kg was injected subcutaneously twice a week for five weeks in total. For prostate regeneration, a pellet of testosterone (20 mg) was implanted under the dorsal skin 8 weeks post-castration. Five weeks later after implantation, mice were sacrificed and the prostate lobes were isolated for histological analysis.

### Ethics statement

All animal work was approved by our Institutional Animal Care and Use Committee in Department of Epigenetics and Molecular Carcinogenesis at the University of Texas M.D Anderson Cancer Center. All animals were maintained in standard conditions according to the Institutional Guidelines. Animal housing rooms were under temperature and humidity control and mice were not subject to water or food restrictions. Laboratory staff monitored mice when surgical techniques were performed. Mice were palpated weekly and monitored daily for tumor determination/assessment. If animals exhibited any indication of tumor burden, sickness, infection or distress, they were administered with appropriate antibiotics, analgesics, or euthanasia.

## SUPPLEMENTARY FIGURES


