# Supplementary material for: Transgenic overexpression of NanogP8 in the mouse prostate is insufficient to initiate tumorigenesis but weakly promotes tumor development in the Hi-Myc mouse model
Source: Oncotarget. 2017 Apr 18;8(32):52746–60. doi: 10.18632/oncotarget.17186 (PMC5581066; doi:10.18632/oncotarget.17186)
Supplement: Supplementary file 1 [file oncotarget-08-52746-s001.pdf]

Transgenic overexpression of NanogP8 in the mouse prostate is insufficient to initiate tumorigenesis but weakly promotes tumor development in the Hi-Myc mouse model

SUPPLEMENTARY FIGURES

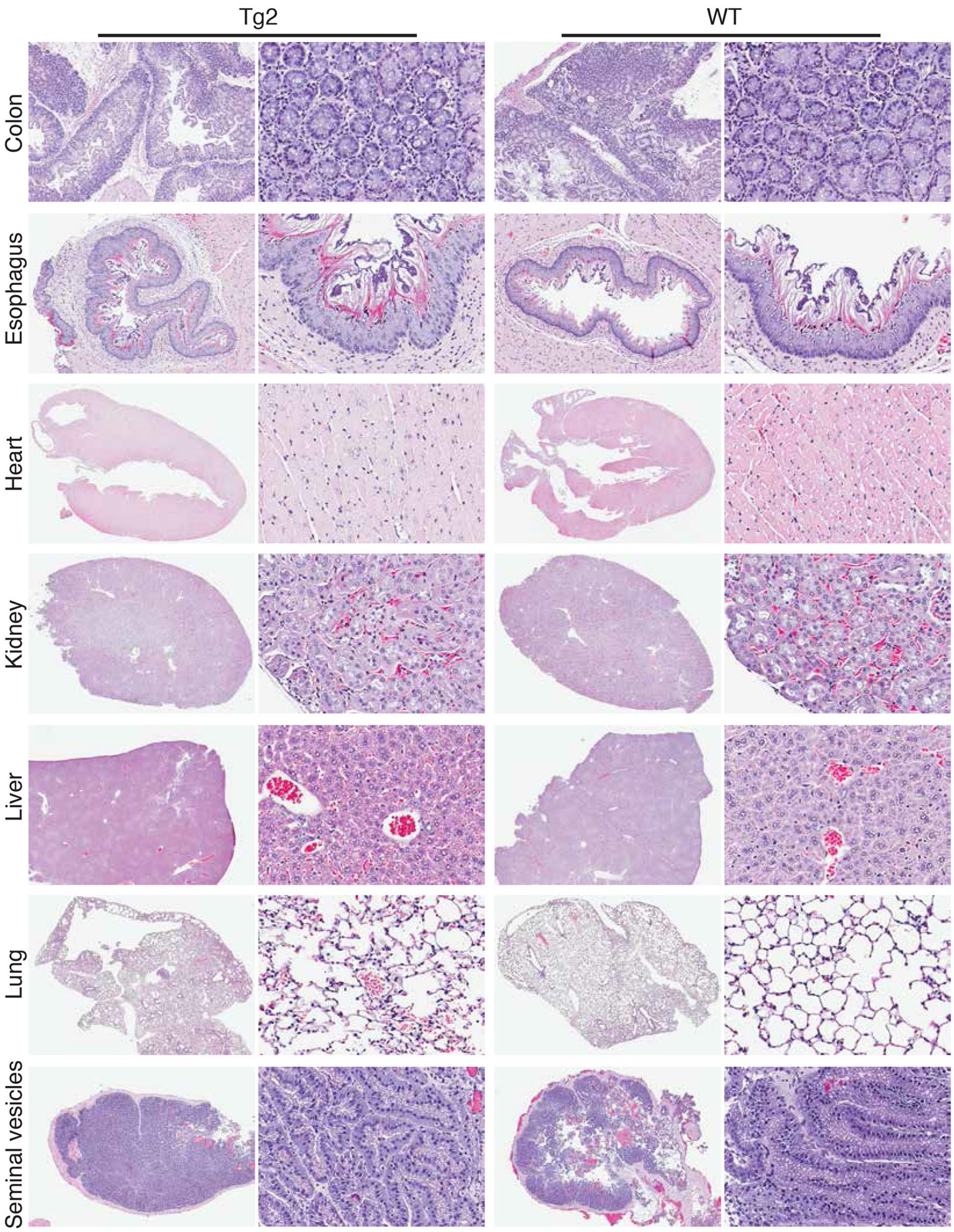

(Continued)

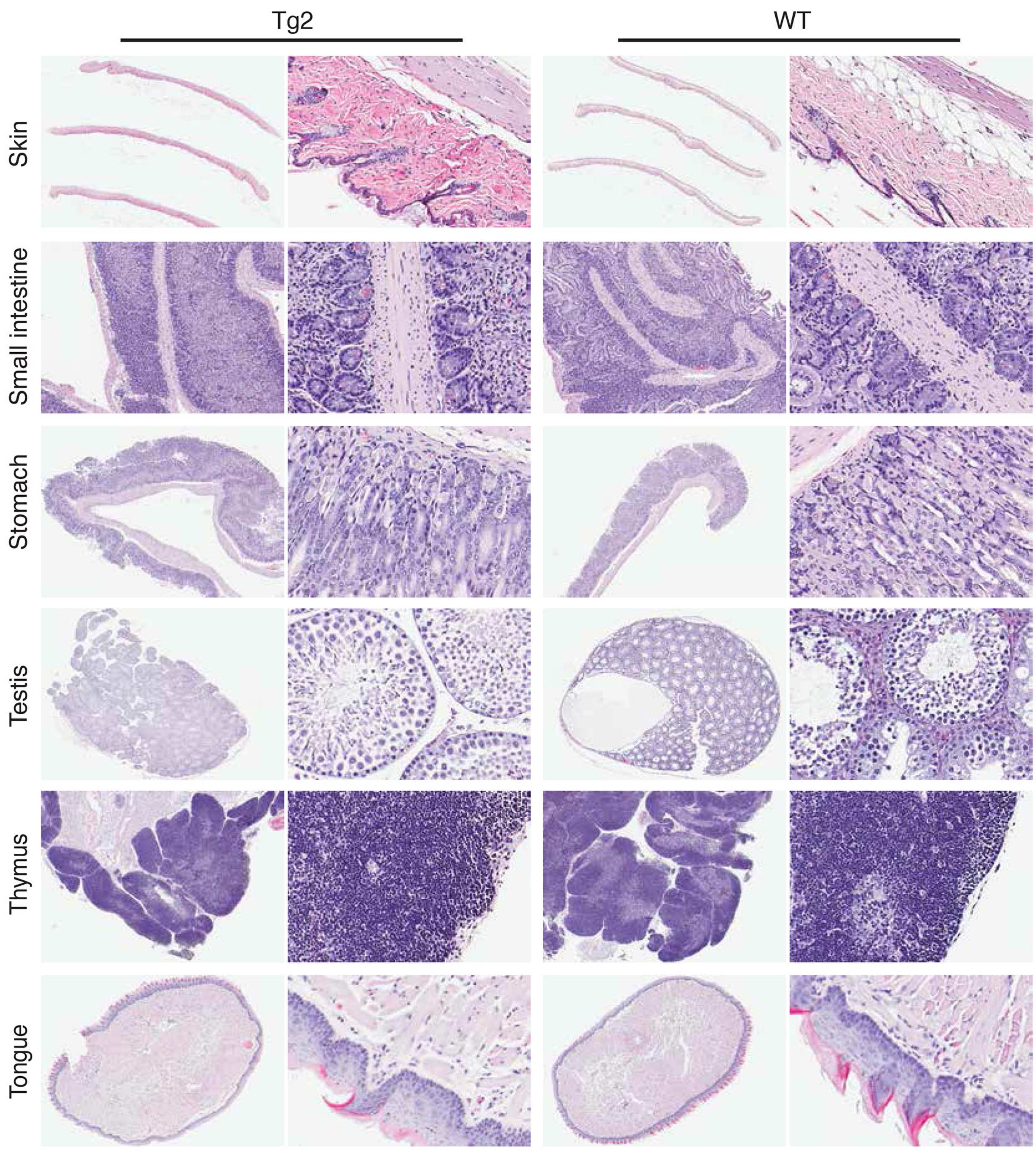

Supplementary Figure 1: Histological characterizations of 13 organs and tissues in WT and Tg2 animals. Shown are H&E images of the indicated tissues/organs of NanogP8 Tg2 and WT mice (~3 months old) including the colon, esophagus, heart, kidney, liver, lung, seminal vesicles, skin, small intestine, stomach, testis, thymus, and tongue. For each organ, a low (40x) and a high (200x) power image were shown.

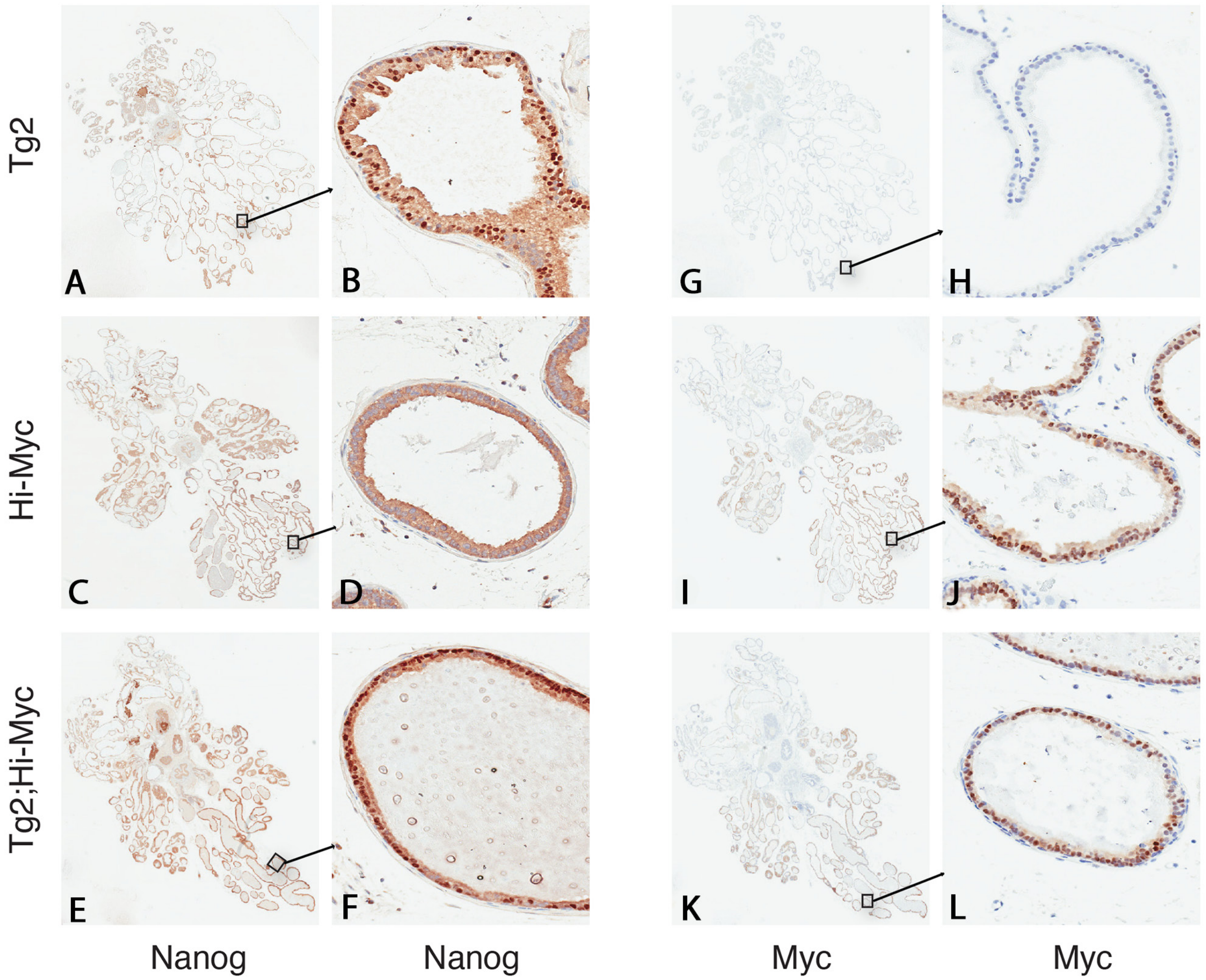

Supplementary Figure 2: Nanog or Myc IHC analysis in the transgenic prostates. The left two panels are the Nanog staining in Tg2, Hi-Myc and Tg2; Hi-Myc mouse prostates. Panels **(A)**, **(C)** and **(E)** are the whole-mount prostate images and panels **(B)**, **(D)** and **(F)** are the magnified VP images (200×). The right two panels represent Myc staining in the 3 transgenic prostates. Panels **(G)**, **(K)** and **(I)** are whole-mount image while panels' **(H)**, **(J)** and **(L)** are VP images with high magnifications (200×).
